# Guestimating Molecular Subtyping of Breast Cancer by Ki67 in the Era of Artificial Intelligence

**DOI:** 10.1155/ijbc/9640277

**Published:** 2026-01-02

**Authors:** Catherine E. Connolly, Barbara Padberg Sgier, Regina Masser, Juliane Friemel, Quentin Simon, Annina Fasler, Eva Karamitopoulou, Marianne Tinguely

**Affiliations:** ^1^ Pathologie Institute Enge, Zürich, Switzerland; ^2^ Institute of Pathology, University Hospital Zürich, Zürich, Switzerland, usz.ch; ^3^ Medical Faculty, Università della Svizzera italiana, Lugano, Canton Ticino, Switzerland, usi.ch; ^4^ Medical Faculty, University of Zürich, Zürich, Switzerland, uzh.ch

**Keywords:** artificial intelligence, breast cancer, endopredict, Ki67, luminal subtyping, Oncotype DX

## Abstract

**Aims:**

This study aimed to compare the performance of immunohistochemistry (IHC)‐based luminal subtyping of breast cancer against gene expression panels at our institute and to evaluate a CE‐certified artificial intelligence (AI) Ki67 image analysis program for improving subtyping accuracy.

**Methods and Results:**

We retrospectively analysed IHC‐based luminal subtyping in breast cancer biopsies diagnosed at our institute from 2019 to 2022 (*n* = 1736), and identified *n* = 104 (Oncotype DX) and *n* = 64 (EndoPredict) cases with gene expression tests requested by clinicians. Of the eligible ER‐positive HER2‐negative cases, 11.9% (*n* = 168) underwent multigene testing. After excluding incomplete data (*n* = 22), gene tests revealed 48 patients (32.9%) would benefit from chemotherapy, 86 (58.9%) could avoid it and 12 (8.2%) had inconclusive results. A moderate correlation was observed between Ki67 and EndoPredict EPClin scores (*r* = 0.47–0.58) and a weak correlation between Ki67 and Oncotype DX recurrence scores (*r* = 0.31–0.38). Ki67 scores were significantly higher in luminal B compared with luminal A tumours (difference of 9.1–15.2, *p* < 0.01). No significant difference was found between mean Ki67 scores reported by pathologists and AI (pathologists’ mean Ki67 17.36 vs. AI mean Ki67 18.36, *n* = 146, *p* = 0.456) and the accuracy of luminal subtyping was similar between pathologists and AI (accuracy pathologists 66.4% vs. AI 62.7%, *p* = 0.538).

**Conclusions:**

Our data provides a snapshot of the real‐world allocation of multigene testing in early breast cancer, and supports other studies in highlighting the discrepancy between IHC‐based and gene‐based luminal subtyping. Ki67 evaluation remained consistent over time, and the use of AI for Ki67 scoring did not enhance the accuracy of IHC‐based luminal subtyping.

## 1. Introduction

With the rising use of gene expression testing in cancer diagnostics, there is a parallel effort to approximate gene‐based diagnoses using surrogate immunohistochemical (IHC) markers. Utilising existing diagnostic biomarkers for genetic approximation offers cost benefits and circumvents the logistical challenges of sending tissue samples internationally. However, this approach carries the risk of misdiagnosis, which can undermine precision medicine. The IHC marker Ki67, used for surrogate luminal subtyping of breast carcinomas, exemplifies the benefits and risks of ‘guestimating’ genetic diagnoses.

In the year 2000, Charles Perou revolutionised the field of breast cancer diagnostics by reclassifying tumour subtypes based on their molecular profiles [1]. Evidence now supports further division of ER+/luminal‐like tumours into luminal A and luminal B subtypes because of their differing survival outcomes and responses to chemotherapy [2–5]. Condensed gene panel studies such as Oncotype DX, EndoPredict, MammaPrint or Prosigna (formerly PAM50) hone in on the most decisive genes for subtyping purposes and achieve this with high accuracy [3]. Meanwhile, Ki67 has emerged as the most significant and widely used IHC marker for making the surrogate distinction between luminal A‐like and B‐like tumours; however, the Ki67 cut‐off for doing so remains elusive [6, 7].

We collated four consecutive years of breast cancer data from our institution, and herein, we scrutinise the performance of Ki67 in surrogate molecular subtyping and explore the impact of artificial intelligence (AI) on delineating luminal tumours, as compared against gene expression test results.

## 2. Material and Methods

### 2.1. Study Population and Data Collection

All core needle biopsies (CNB) demonstrating B5b invasive breast carcinoma over a period of 4 years at our institution (2019–2022, *n* = 1736) were identified using our laboratory informatics system PathoWin+ (v1.10.4.38642, Basys Data GmbH, Basel, Switzerland). There were no exclusion criteria, and baseline categorical and numerical variables were obtained from validated pathology reports. The variables of interest included the following: age, gender, histological tumour type, tumour grade, IHC markers (ER, PR, HER2 and Ki67), in‐situ hybridisation (ISH) HER2 status and Oncotype DX (*n* = 104) or EndoPredict (*n* = 64) results where available. ER and PR were considered positive if ≥ 1% of cells stained positively by IHC. HER2 was assessed by IHC and ISH as per the 2018 ASCO/CAP guidelines and Ki67 was quantified as a percentage expression following tissue staining with CONFIRM Ki‐67 (30‐9) rabbit monoclonal primary antibody (Roche Diagnostics, Switzerland) [8]. Oncotype DX and EndoPredict testing were carried out upon request by clinicians and in accordance with local guidelines. Information extracted from the Oncotype DX reports included the following: Recurrence Score (RS), ER, PR, HER2 status and comments on the benefit or otherwise of chemotherapy. From the EndoPredict reports, we considered the 12‐gene molecular (EPgene) and combined molecular and clinical (EPclin) scores. For the Oncotype DX and EndoPredict cohorts, the original Ki67 biopsy slides were retrieved and re‐scored by three practicing breast pathologists and by AI. Ethical approval was granted by the Ethics Committee of the Canton of Zurich (BASEC‐Nr.: 2023‐01822).

### 2.2. Molecular Subtyping by Gene Studies

Classification of luminal subtypes by gene studies was performed using the results of either Oncotype DX or EndoPredict tests. Oncotype DX tests the expression of 16 tumour‐associated genes and five reference genes and provides prognostic and predictive information for patients with early‐stage, ER+/HER2− breast cancer. The major cancer‐related gene categories tested by Oncotype DX include the following: proliferation, invasion, HER2, oestrogen and other [3]. For Oncotype DX cases in which chemotherapy was recommended, we assigned luminal B status and for cases in which chemotherapy was negated, we assigned luminal A status.

EndoPredict tests five hormone receptor‐ and three proliferation‐associated genes, in addition to four control genes, to provide the EPgene score. Additionally, and in contrast to Oncotype DX, the EndoPredict test also requires the input of further tumour‐ and patient‐related characteristics, which are combined with the gene results to provide the EPclin score [3]. For EndoPredict, we defined luminal subtypes according to their EPgene or EPclin score, with cut‐offs of 5 and 3.3, respectively.

### 2.3. Surrogate Molecular Subtyping by Immunohistochemistry

Classification of luminal‐like subtypes by IHC was performed in accordance with the 2013 St. Gallen International Breast Cancer Consensus: Luminal A‐like (ER‐positive, PR‐positive, HER2‐negative, Ki67 low); Luminal B‐like (ER‐positive, HER2‐negative and at least one of: Ki67 high and/or PR negative or low) [9]. Unless otherwise specified, a threshold of ≥ 20% was used to define high Ki67. For the purpose of surrogate luminal subtyping, the PR threshold was set at ≥ 20%, as derived by the work of Prat et al. and recognised by the St. Gallen Consensus [9, 10]. IHC‐based subtyping was performed using biopsy specimens and in addition to analysing the Ki67 value in the original report, three pathologists rescored the Ki67 for study purposes.

### 2.4. Ki67 Quantification by Artificial Intelligence

**Figure 1:**
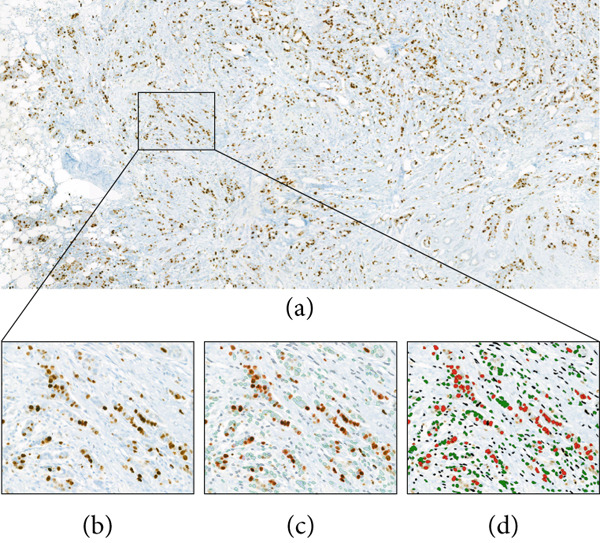
Overview of workflow using VMScope Ki67 Quantifier: (a) Whole slide image captured by slide scanner, followed by (b) user‐defined selection of representative area for quantification at 20× magnification. The quantifier provides (c) outline or (d) overlay images demonstrating the cells that are identified as positive tumour cells (red), negative tumour cells (green) and non‐tumour cells (black). A Ki67 percentage for each area, and an average for all selected areas, are provided.

Original Ki67 IHC‐slides were retrieved from the laboratory archive and digitalised using the Pannoramic 250 Flash III Scanner (3DHISTECH, Sysmex, Switzerland). Images were stored and viewed in CaseViewer v2.4 (3DHISTECH, Sysmex, Switzerland). The CognitionMaster Professional Suite Ki67 Quantifier (v5.1.8124.25467, VMScope, Berlin, Germany) was used to determine Ki67 expression at 20× magnification, using standardised brightness and field size selection between slides (Figure 1). One pathologist selected three representative areas of tumour per slide, and the process was repeated following a washout period of 2 weeks.

### 2.5. Statistical Analyses

Statistical analyses were performed in RStudio version 1.4.1103 for Windows (RStudio, Posit Software) and PSPP statistics version 1.4.1 (GNU, Free Software Foundation). Frequency distribution curves of log‐transformed Ki67 values were performed following the methodology of Wolf et al. [8]. The concordance of IHC markers with corresponding Oncotype DX results was measured with Cohen’s *Κ*. Spearman’s correlation coefficient was calculated for the relationship between Ki67 and Oncotype DX RS or EndoPredict EPgene and EPclin scores [11]. Welch’s *t* test was used to calculate the difference in geometric mean Ki67 between study cohorts following log transformation to satisfy normal distribution. All presented mean Ki67 values are geometric, unless otherwise specified. Sensitivity, specificity, accuracy and positive and negative predictive values were calculated for the performance of IHC‐based surrogate luminal subtyping against results of gene studies. The optimal Ki67 cut‐off percentage to discriminate between luminal A and B groups was determined using receiver operating characteristic (ROC) curve analyses. *p* values of < 0.05 were considered statistically significant.

## 3. Results

### 3.1. Cohort Summary

A total of 1736 patients who underwent CNB of the breast showing B5b invasive carcinoma during the years 2019–2022 were included in this study. The average patient age was 62 years, and all but 18 were female. The distribution of histological subtypes included 78.9% invasive NST, 16.2% lobular carcinomas and the remainder were classified as ‘other’. The most common tumour grade was 2 (48.2%), followed by Grade 3 (31.5%) and Grade 1 (19.9%). Our HER2 positivity rate was 12.3% and a total of 172 triple negative carcinomas were diagnosed (9.9%). The median Ki67 of the entire cohort was 10% (range 1%–100%) and the mean was 13.4%. There were 1414 (81.5%) luminal‐like tumours (ER‐positive/HER2‐negative) within our study cohort. Table 1 provides a summary of cohort characteristics.

**Table 1:** Tumour characteristics of patients with B5b biopsy results, 2019–2022.

**Number of tumours (%)**					
	**All years**	**2019**	**2020**	**2021**	**2022**
No. of cases	1736	387	412	510	427
Tumour type					
Ductal	1370 (78.9)	317 (81.9)	327 (79.4)	387 (75.9)	339 (79.4)
Lobular	281 (16.2)	59 (15.3)	64 (15.5)	91 (17.8)	67 (15.7)
Other	85 (4.9)	11 (2.8)	21 (5.1)	32 (6.3)	21 (4.9)
Tumour grade					
1	345 (19.9)	80 (20.7)	92 (22.3)	89 (17.5)	84 (19.7)
2	837 (48.2)	182 (47)	198 (48.1)	247 (48.4)	210 (49.2)
3	546 (31.5)	125 (32.3)	121 (29.4)	172 (33.7)	128 (30)
ER					
+	1487 (85.7)	333 (86.1)	352 (85.4)	434 (85.1)	368 (86.2)
−	249 (14.3)	54 (14)	60 (14.6)	76 (14.9)	59 (13.2)
PR					
+	1307 (75.3)	300 (77.5)	311 (75.5)	389 (76.3)	307 (71.9)
−	428 (24.7)	86 (22.2)	101 (24.5)	121 (23.7)	120 (28.1)
HER2 IHC					
0	667 (38.4)	178 (46)	159 (38.6)	185 (36.3)	145 (34)
1+	572 (32.9)	115 (29.7)	131 (31.8)	161 (31.6)	165 (38.6)
2+	328 (18.9)	60 (15.5)	79 (19.1)	116 (22.8)	73 (17.1)
3+	168 (9.7)	34 (8.8)	42 (10.2)	48 (9.4)	44 (10.3)
HER2 ISH					
+	163 (9.4)	23 (5.9)	26 (6.3)	61 (12)	53 (12.4)
−	286 (16.5)	42 (10.9)	76 (18.5)	102 (20)	66 (15.5)
HER2 Status					
+	213 (12.3)	51 (13.2)	48 (11.7)	61 (12)	53 (12.4)
−	1520 (87.6)	335 (86.6)	363 (88.1)	448 (87.8)	374 (87.6)
Triple Neg.	172 (9.9)	33 (8.5)	41 (10)	55 (10.8)	43 (10.1)
Ki67, %					
Median	10	10	10	10	15
Geometric mean	13.4	13.4	13.1	13.2	14
Arithmetic mean	22.1	21.4	20.1	22.2	24.5
Range	1–100	2–95	2–95	1–95	1–100

### 3.2. Comparison of Surrogate Luminal Subtyping Against Results of a 21‐Gene Panel

We identified all cases in our study cohort which underwent Oncotype DX testing and for which the biopsy and resection specimens were both processed at our institute (*n* = 98). The characteristics of these cases are summarised in Table 2.

**Table 2:** Characteristics of patients with B5b tumours and Oncotype DX or EndoPredict gene test results, 2019–2022.

	**Oncotype DX**	**EndoPredict**
No. of cases	98	48
Average patient age, years	58	58
Range	28‐76	40‐81
Tumour type, no. (%)		
Ductal	83 (84.7)	42 (87.5)
Lobular	11 (11.2)	6 (12.5)
Other	4 (4.1)	0
Tumour grade, no. (%)		
1	9 (9.2)	9 (18.8)
2	54 (55.1)	29 (60.4)
3	35 (35.7)	10 (20.8)
Average tumour size, mm	23	23.5
Range	8‐85	5‐50
Nodal status, no. (%)		
0	42 (42.9)	23 (47.9)
1mi	8 (7.7)	6 (12.5)
1a	42 (42.9)	18 (37.5)
2	0	1 (2.1)
Tumour invasion, no. (%)		
Lymph	54 (55.1)	26 (54.2)
Vascular	3 (3.1)	2 (41.7)
Perineural	26 (26.5)	11 (22.9)
ER, no. (%)		
+	98 (100)	48 (100)
−	0 (0)	0 (0)
PR, no. (%)		
+	87 (88.8)	45 (93.8)
−	11 (11.2)	3 (6.3)
HER2 IHC, no. (%)		
0	44 (44.9)	11 (22.9)
1+	38 (38.8)	23 (47.9)
2+	16 (16.3)	14 (29.2)
3+	0	0
HER2 ISH, no.		
+	0	0
−	16	14
HER2 status, no.		
+	0	0
−	98 (100)	48 (100)
Ki67, %		
Median	15/25^a^	15/20^a^
Geometric mean	15.4/21.8^a^	14/19.1^a^
Arithmetic mean	19.1/27^a^	17.8/23.1^a^
Range	3‐90	5‐70
Gene test result, no. (%)		
Luminal A	67 (68.4)	14/19 (29.2/39.6)^b^
Luminal B	19 (19.4)	34/29 (70.8/60.4)^b^
Uncertain	12 (12.2)	na

^a^Biopsy/resection.

^b^EPgene/EPclin result.

We retrospectively observed that the mean Ki67 for our Oncotype DX‐determined luminal B cohort was 10.3% higher than the mean Ki67 for our luminal A cohort (luminal B 23.7% vs. luminal A 13.4%, *p* < 0.01). In addition to supporting or negating the use of chemotherapy, Oncotype DX also identifies an ‘uncertain’ group, in which the benefit of chemotherapy ‘cannot be excluded’ and the mean Ki67 for this group in our cohort was 16.2%. The distribution of our 98 cases according to Oncotype DX included 67 luminal A (68.4%), 12 ‘uncertain’ (12.2%) and 19 luminal B (19.4%) tumours. However, had we relied solely on performing IHC‐based luminal‐like subtyping using the 2013 St. Gallen Consensus Ki‐67 cut‐off of 20%, we would have observed a shift to a more or less equal distribution of both cohorts with 51 luminal A‐like (52%) and 47 luminal B‐like (48%) tumours. Retrospective IHC‐based subtyping enabled the correct classification of 43/67 (64.2%) luminal A and 16/19 (84.2%) luminal B tumours (Figure 2).

Figure 2Sankey diagrams showing IHC‐based surrogate luminal subtyping results using a Ki67 cut‐off of 20% in comparison with (a) Oncotype DX or (b) EndoPredict EPclin results (luminal A = green, luminal B = orange, uncertain = yellow). The diagrams provide a visual overview of the number of cases where the IHC‐based classification (shown on the left side) was different to the multigene test‐based classification (shown on the right side) by means of crossing colour strands.

**Figure:**
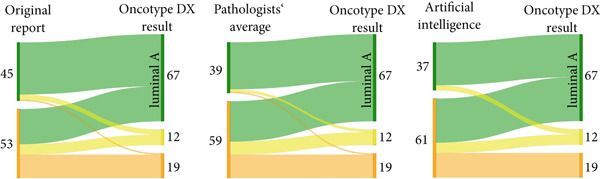
(a)

**Figure:**
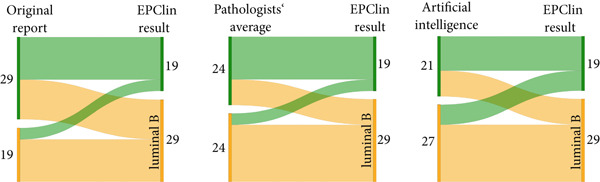
(b)

For our other routine IHC markers, we observed 100% agreement in ER status, and 94.9% agreement in HER2 status with the Oncotype DX results. Three of five discordant HER2 cases were reported as IHC 2+, negative by ISH and ‘borderline’ by Oncotype DX, and the remaining 2/5 cases were IHC 1+, did not undergo ISH and were ‘borderline’ according to Oncotype DX. There was a *weak correlation* between Ki67 and Oncotype DX RS (*r* = 0.31, *p* < 0.01).

### 3.3. Comparison of Surrogate Luminal Subtyping Against Results of a 12‐Gene Panel

Within our study cohort, we also identified *n* = 48 cases that were investigated with EndoPredict, the characteristics of which are presented in Table 2. There was no significant difference in patient age or tumour size compared with the Oncotype DX cohort. When stratified by EPclin score into luminal A and luminal B groups, we observed a 9.3% difference in mean Ki67 scores (luminal A mean Ki67 9.2% vs. luminal B mean Ki67 18.5%, *p* < 0.01). Ki67 scores correlated *moderately* with EPclin scores (*r* = 0.47, *p* < 0.01) as well as with EPgene scores (*r* = 0.59, *p* < 0.01).

We noted seven cases with high EPgene scores, which were re‐classified as low risk by EPclin score—the Ki67 scores for these cases were 7% (*n* = 3), 8% (*n* = 1), 15% (*n* = 2) and 25% (*n* = 1). Meanwhile there were two cases with low EPgene scores, which were re‐classified as high risk by EPclin score—the Ki67 scores for both cases were 10%. Overall, EPclin scores classified 19 tumours as low‐risk (luminal A, 39.6%) and 29 tumours as high risk (luminal B, 60.4%). However, had we relied on IHC‐based subtyping with a Ki67 20% cut‐off alone, we would have observed the inverse distribution of luminal A‐like and B‐like tumours. Retrospective IHC‐based classification enabled the correct classification of 15/19 (78.9%) luminal A and 13/29 (44.8%) luminal B tumours (Figure 2).

### 3.4. Artificial Intelligence and Consensus Ki67 Scoring for Luminal‐Like Subtyping

All original Ki67 biopsies (*n* = 146) from the Oncotype DX and EndoPredict cohorts were independently re‐scored by three practicing breast pathologists, and digitalised for assessment by AI. There was no significant difference in the mean Ki67 scoring between pathologists and AI (pathologists mean Ki67 17.36% vs. AI mean Ki67 18.36%, *p* = 0.456). The correlation between Oncotype DX RS and Ki67 as assessed by AI (*r* = 0.38) and pathologists (*r* = 0.33) was similar (*p* = 0.347); as was the correlation between EndoPredict EPgene score and Ki67 according to AI (*r* = 0.60) and pathologists (*r* = 0.65, *p* = 0.348). A summary of Ki67 scoring, and the performance of IHC‐based luminal subtyping using various Ki67 scoring methods is shown in Tables 3 and 4.

**Table 3:** Geometric mean Ki67 scores (%) for gene expression test‐determined luminal subtypes (*n* = 134).

	**Original report**	**Pathologists’ average**	**Artificial intelligence**
Luminal A	12.37	13.31	15.64
Luminal B	20.4	26	23.58

**Table 4:** Performance of IHC‐based surrogate luminal subtyping in comparison with gene expression test results, using Ki67 values determined by the original report, the average of three pathologists, or artificial intelligence.

	**Original report**	**Pathologists’ average**	**Artificial intelligence**
Sensitivity (%)	63.95	59.30	52.33
Specificity (%)	68.75	79.17	81.25
PPV (%)	78.57	83.61	83.33
NPV (%)	51.56	52.05	48.75
Accuracy (%)	65.67	66.42	62.69

The overall accuracy of IHC‐based classification was the highest using the mean Ki67 of three pathologists (accuracy 66.42%). However, this did not significantly differ from accuracies using the original report (65.67%) or AI‐based (62.69%) Ki67 scores. In addition, we investigated the optimal Ki67 cut‐offs using each scoring method to determine which cut‐offs achieved the best concordance in luminal subtyping as compared against multigene panels. Results are shown in ROC curves in Figure 3, paired with box plots of Ki67 scoring distributions for each method. The applied St. Gallen recommended Ki67 cut‐off of 20% was confirmed to be optimal for AI‐based Ki67 assessment. If Ki67 cut‐offs of ≤ 5% or ≥ 30% are applied, as per the Ki67 Working Group Recommendations, then 83/146 tumours (56%, pathologists) or 114/146 tumours (78%, AI) remain unclassified [7].

Figure 3ROC curves showing the performance of IHC‐based surrogate luminal subtyping using a Ki67 cut‐off of 20% against results of gene tests; and distribution plots of Ki67 values for luminal A and B tumours. The maximal AUC and optimal Ki67 cut‐off are shown for (a) Oncotype DX and (b) EndoPredict (EPclin) cohorts. The distribution plots show a trend for higher Ki67 values in luminal B compared with luminal A tumours.

**Figure:**
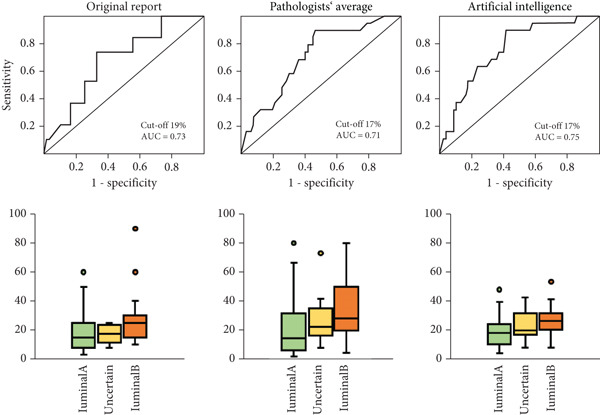
(a)

**Figure:**
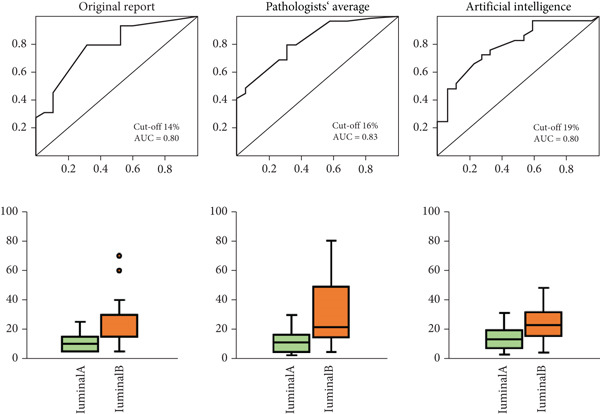
(b)

## 4. Discussion

In this study, we retrospectively analysed the performance of our institute’s IHC‐based surrogate luminal subtyping, using ER, PR, HER2 and Ki67, against available results from the gene expression assays, Oncotype DX and EndoPredict, which were performed according to clinician requests in a real‐world context. We found that 11.9% of our eligible ER‐positive, HER2‐negative B5b breast cancer cohort over a 4‐year period underwent gene expression testing. Our findings support the observation of other studies, showing that IHC‐based and gene expression‐based luminal subtypes do not reliably define the same patient cohorts [12–14]. The limited accuracy of IHC‐based surrogate luminal subtyping could not be harmonised in our cohort by using laboratory‐specific Ki67 cut‐offs or an artificial intelligence aid for Ki67 scoring.

Our cohort characteristics are comparable with a 4‐year observational study of predictive markers from a tertiary referral centre in Switzerland [15]. When compared against accepted worldwide averages, we observed slightly lower rates of triple negative (8.5%–10.8%) and HER2‐positive (11.7%–13.2%) tumours [16]. Ethnicity or socioeconomic data were not collected for our cohort, which are two recognised factors that may affect the incidence of certain breast cancer subtypes [17]. Overall, our cohort demonstrated stable tumour typing, grading and receptor expression patterns across the study period.

Ki67 scores taken from the original report at the time of diagnosis were found to be significantly higher in both Oncotype DX‐ (10.3% higher) and EndoPredict‐diagnosed (9.3% higher) luminal B tumours in comparison with luminal A (both *p* < 0.05). In this study, we attempted to implement artificial intelligence scoring of Ki67 to harmonise the discrepancies in IHC‐ and gene‐based subtyping. However, we observed no significant differences in comparison with conventional scoring or consensus pathologist scoring. Even after performing ROC curves and applying our calculated AI‐specific Ki67 cut‐off, we did not observe improvements in luminal subtyping accuracy.

By applying the Ki67 Working Group recommendation that only Ki67 scores ≤ 5% or ≥ 30% should be used for subtyping, then depending on the scoring method applied, up to 78.1% of tumours would remain unclassified because of their middle‐range Ki67 scores [7]. Our cohort illustrates this key pitfall associated with implementing the best‐practice guidelines for surrogate subtyping, particularly when gene expression tests may not be readily available or affordable for patients [7, 9].

Upon further breakdown analyses, we noted that Oncotype DX, after exclusion of inconclusive results, revealed 67 luminal A (77.9%) and 19 luminal B (22.1%) tumours, whereas EndoPredict revealed 19 luminal A (39.6%) and 29 luminal B (60.4%) tumours. Further investigation of the significant difference in overall ratio of luminal A and B tumours detected by these tests lay outside of the scope of this study, but has been alluded to in other works where 18% of cases showed a major discrepancy when tissue was submitted for both tests [18]. Another study comparing EndoPredict and MammaPrint found similar proportions of luminal A (43%–45%) and B (55%–57%) tumours detected by each test, but there were contradictory predictions for the same tumour in over a third of cases (36%) [19].

Contrary to our cohort, the ABCSG‐6/8 validation cohorts for EndoPredict detected more low‐risk (*n* = 1066, 62.6) than high‐risk (*n* = 636, 37.4%) tumours [20]. Possible factors explaining the difference in observed ratios, include that the combined ABCSG‐6/8 cohort had more node‐negative patients (*n* = 1165, 68%) and more tumours ≤ 2 cm (*n* = 1136, 67%) compared with our cohort (node‐negative patients: *n* = 23, 48%; tumours ≤ 2 cm: *n* = 21, 44%) [20].

In our study, it is important to note that the selection of patients for gene testing occurred by means of case discussions in multidisciplinary tumour board meetings. Therefore, our cohort predominantly consists of patients whose conventional prognostic markers were not clearly suggestive for or against the use of chemotherapy, which may in part explain the discrepancy we observed between IHC‐signatures and gene expression test results. Tan et al. similarly concluded that where there is no clear clinicopathological indication for or against chemotherapy, Ki67 is not a substitute for Oncotype DX testing [14]; and Denkert et al. argue that “clinical decisions should not be based on small differences in the intermediate range” of Ki67, and that “we should probably stop looking for an optimal cutpoint for Ki67 because it simply does not exist” [21].

A limitation of our work is that we did not investigate different Ki67 scoring techniques (e.g., average vs. hotspots, counting vs. eyeballing), which are known to influence the utility of this biomarker. These topics have been previously addressed by other studies and remain contentious [7, 22, 23]. A further limitation to this work is the lack of long‐term outcomes data (e.g., recurrence, survival) to verify the performance of gene tests against IHC‐based subtyping.

In conclusion, our study highlights that AI does not help to resolve critical aspects of Ki67 as a prognostic and predictive surrogate marker for gene expression profiling in early breast cancer. Our cohort depicts IHC‐based luminal‐like subtyping and gene‐expression‐based luminal subtyping as two distinct cohorts, and cautions researchers and clinicians on the pitfalls of institute‐specific Ki67 cut‐offs.

## Ethics Statement

Ethical approval was granted by the Ethics Committee of the Canton of Zurich (BASEC‐Nr.: 2023‐01822).

## Conflicts of Interest

The authors declare no conflicts of interest.

## Author Contributions

Conceptualization and methodology: Catherine E. Connolly, Marianne Tinguely; Formal analysis and investigation: Annina Fasler, Barbara Padberg Sgier, Catherine E. Connolly, Eva Karamitopoulou, Juliane Friemel, Marianne Tinguely, Regina Masser, Quentin Simon; Dara curation: Catherine E. Connolly, Marianne Tinguely; Writing – original draft preparation: Barbara Padberg Sgier, Catherine E. Connolly, Marianne Tinguely; Writing – review and editing: Barbara Padberg Sgier, Catherine E. Connolly, Eva Karamitopoulou, Juliane Friemel, Marianne Tinguely, Regina Masser, Quentin Simon; Supervision: Marianne Tinguely.

## Funding

No funding was received for this manuscript.

## Data Availability

Data will be made available from the corresponding author upon reasonable request.
